# Caregivers’ Role in In-Home Video Telehealth: National Survey of Occupational Therapy Practitioners

**DOI:** 10.2196/52049

**Published:** 2024-03-14

**Authors:** Megan Elizabeth Gately, Dylan E Waller, Emily E Metcalf, Lauren R Moo

**Affiliations:** 1 VA Bedford Health Care System Geriatric Research Education and Clinical Center (GRECC) Bedford, MA United States; 2 Boston University School of Medicine Division of Geriatrics Boston, MA United States; 3 VA Portland Health Care System Center to Improve Veteran Involvement in Care (CIVIC) Portland, OR United States; 4 Oregon Medical Research Center Portland, OR United States; 5 Massachusetts General Hospital Cognitive Behavioral Neurology Unit Boston, MA United States

**Keywords:** telemedicine, caregivers, occupational therapy, caregiver, care worker, telehealth, older adults, older adult, geriatric, rural, remote, OT practitioner, web-based, national survey, role, home care, clinical support, mobile phone

## Abstract

**Background:**

Older adults face barriers to specialty care, such as occupational therapy (OT), and these challenges are worse for rural older adults. While in-home video telehealth may increase access to OT, older adults’ health- and technology-related challenges may necessitate caregiver assistance.

**Objective:**

This study examines caregiver assistance with in-home OT video telehealth visits from the perspectives of OT practitioners at Veterans Health Administration (VHA).

**Methods:**

A web-based national survey of VHA OT practitioners about caregivers’ role in video telehealth was conducted between January and February 2022. Survey items were developed with input from subject matter experts in geriatrics and OT and identified patient factors that necessitate caregiver participation; the extent to which caregivers assist with different types of tasks (technological and clinical tasks); and the perceived facilitators of, benefits of, and barriers to caregiver involvement.

**Results:**

Of approximately 1787 eligible VHA OT practitioners, 286 (16% response rate) participated. Not all survey items required completion, resulting in different denominators. Most respondents were female (183/226, 81%), White (163/225, 72.4%), and occupational therapists (275/286, 96.2%). Respondents were from 87 VHA medical centers, the catchment areas of which served a patient population that was 34% rural, on average (SD 0.22). Most participants (162/232, 69.8%) had >10 years of OT experience serving a patient cohort mostly aged ≥65 years (189/232, 81.5%) in primarily outpatient rehabilitation (132/232, 56.9%). The top patient factors necessitating caregiver involvement were lack of technical skills, cognitive impairment, and advanced patient age, with health-related impairments (eg, hearing or vision loss) less frequent. Technological tasks that caregivers most frequently assisted with were holding, angling, moving, repositioning, or operating the camera (136/250, 54.4%) and enabling and operating the microphone and setting the volume (126/248, 50.8%). Clinical tasks that caregivers most frequently assisted with were providing patient history (143/239, 59.8%) and assisting with patient communication (124/240, 51.7%). The top facilitator of caregiver participation was clinician-delivered caregiver education about what to expect from video telehealth (152/275, 55.3%), whereas the top barrier was poor connectivity (80/235, 34%). Increased access to video telehealth (212/235, 90.2%) was the top-rated benefit of caregiver participation. Most respondents (164/232, 70.7%) indicated that caregivers were at least sometimes unavailable or unable to assist with video telehealth, in which case the appointment often shifted to phone.

**Conclusions:**

Caregivers routinely assist VHA patients with in-home OT video visits, which is invaluable to patients who are older and have complex medical needs. Barriers to caregiver involvement include caregivers’ challenges with video telehealth or inability to assist, or lack of available caregivers. By elucidating the caregiver support role in video visits, this study provides clinicians with strategies to effectively partner with caregivers to enhance older patients’ access to video visits.

## Introduction

### Background

Providing care to 9 million veterans across 1321 facilities, Veterans Health Administration (VHA) is the largest integrated health care system in the United States [1]. A large portion of veterans served are classified as living in rural areas [2], with more than half of VHA enrollees traveling >25 miles to access care [3]. Patients living in the rural United States face difficulties accessing health care that are distinct from their urban counterparts. This is partly due to geography, as physician practices, hospitals, and other health care delivery resources are primarily situated in urban areas. For example, one-sixth of rural residents live 35% further away from an intensive care hospital than urban residents [4]. These disparities are even more striking when factoring in socioeconomic status. As public transit options in rural areas are often limited or nonexistent, patients who do not own reliable means of transportation face additional travel barriers. When comparing low-income rural and urban individuals, low-income rural individuals face worse health outcomes [5].

Disparities are further compounded by other sociodemographic factors. Rural Black people experience poorer health outcomes than their White counterparts [6,7], potentially because of social and environmental factors [8]. Patient age is also a factor when considering the impact of rurality on health, as the proportion of adults aged >65 years living in rural areas (17.5%) is larger than that living in urban areas (13.8%), with the divide expected to increase as the population ages [9]. Geriatric care is difficult to access for rural individuals, as 90% of geriatric physicians practice in urban areas [10]. Furthermore, older adults are more likely to have complex medical needs (eg, multiple chronic conditions and increased rates of dementia or disability), which can lead to an increased risk for institutionalization and the necessity for specialty care services.

One such specialty service is occupational therapy (OT), which assists older adults to age in place by supporting them to participate in meaningful activities ranging from activities of daily living, such as dressing or bathing [11,12], to leisure and work activities [13]. OT has been demonstrated to reduce older adult fall risk and increase older adult safety through home modifications [14], strength training, and educational interventions [15]. OT practitioners work with older adults with complex challenges, such as low vision and Alzheimer disease and related dementias, and frequently work with caregivers [16-19]. Similar to geriatrics and other specialty health care services, there are fewer OT practitioners in rural areas (2 per 10,000) versus urban areas (3 per 10,000) [20]. Ironically, the complex medical needs that necessitate OT services often make traveling to appointments with OT practitioners difficult.

Video telehealth is one of the ways to increase access to specialty services, such as OT; however, older adults may face barriers to video telehealth. Video telehealth expansion during the COVID-19 pandemic allowed clinicians, such as OT practitioners, to deliver rehabilitation services into patients’ homes [21-23], thus increasing access by those for whom distance was a barrier [24]. However, although in-home video telehealth is ideal for OT, which focuses care delivery on the intersection between the person and the environment [25], there may be unique considerations for in-home OT video telehealth with older adults. For example, many older adults face challenges with technology due to age, health-related impairments, or low technical literacy [26]. OT practitioners may also want to see the home environment, and ambulating through the home while holding a video-enabled device may be challenging for older adults with mobility challenges. Furthermore, communication via video sessions may be more challenging for older adults with hearing or cognitive impairment. Caregivers may bridge the divide between older adults and in-home video telehealth. However, our recent scoping review of caregiver involvement in OT in-home video telehealth found little research examining caregivers’ support role [27]. Given the breadth of OT services, which may involve hands-on provision of care and an emphasis on visualizing the patient and the environment, understanding the caregiver support role in OT video visits has potential applicability to myriad medical services delivered via video sessions by a range of clinician disciplines.

### Objectives

To address this knowledge gap, this study examined the caregiver’s role in supporting patient engagement in in-home video telehealth visits for OT services from the perspectives of VHA OT practitioners. Specifically, we sought to identify patient factors that necessitate caregiver participation in in-home OT video telehealth encounters; the extent to which caregivers assist with different types of tasks (technological and clinical tasks); and the perceived facilitators of, benefits of, and barriers to caregiver involvement.

## Methods

### Participants

**Figure 1:**
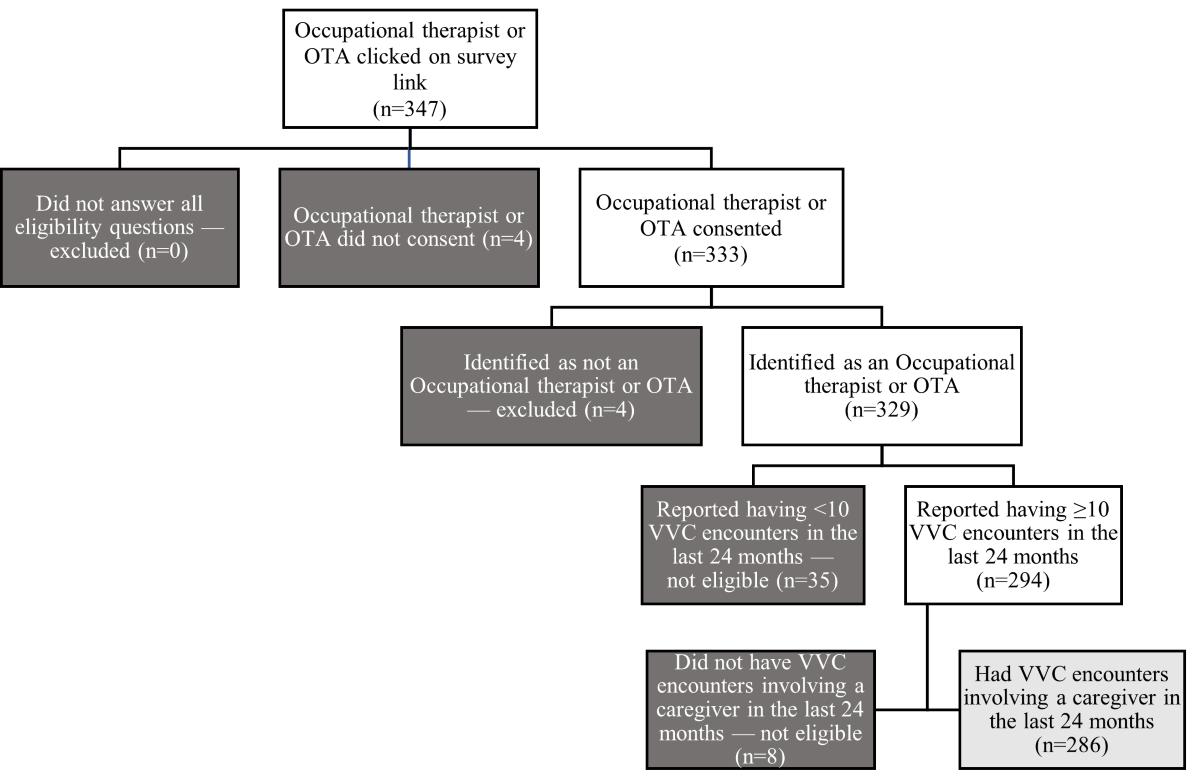
Participant flow diagram. OTA: occupational therapy assistant; VVC: VA Video Connect.

A national survey was conducted with a volunteer sample of VHA OT practitioners (occupational therapists and OT assistants [OTAs]). From approximately 1787 OT practitioners employed across (at the time of survey administration) 1284 health care facilities (171 Veterans Affairs [VA] medical centers and 1113 outpatient sites) during the recruitment period, 333 (18.63%) consented to participate, and 286 (16% response rate) met the eligibility requirements and were included in the study (refer to Figure 1 for the survey flow). The criteria for participation included (1) being an occupational therapist or OTA and (2) having completed at least 10 in-home video telehealth encounters using VA Video Connect (VVC), VHA’s proprietary videoconferencing software, involving a caregiver in the 24 months preceding the survey launch. No other eligibility criteria were applied.

### Survey Development

Here, we outline the survey details guided by the Checklist for Reporting Results in Internet E-Surveys [28]. Survey items gathered information about OT practitioners’ demographics, including practice settings and populations served; patient factors that necessitated caregiver participation in in-home video telehealth; facilitators of caregiver participation; caregiver assistance with both technological and nontechnological tasks; the benefits of and barriers to caregiver involvement; and caregiver availability and relationship to the patient.

Initial survey items were developed in consultation with 7 subject matter experts (SMEs) in geriatrics, OT, caregiver concerns, and survey methodology. In addition to this collective experience, the development of survey items was informed by 2 sources. First, we drew on data regarding caregiver involvement in video telehealth gathered from interviews conducted between January and April 2021 with OT practitioners who were frequent users of in-home video telehealth. The interviews broadly discussed OT practitioners’ use of video telehealth and included questions about caregivers’ support role. Analysis of interview data related to caregiver involvement in video telehealth [29] informed the development of survey items. Specifically, the interview results that informed survey items were those about (1) patients for whom caregivers tended to be involved in video appointments, which informed the survey item about patient factors contributing to caregiver involvement; (2) what caregivers did during video telehealth visits, which informed survey items about the technological and clinical tasks with which caregivers assisted; and (3) how caregiver involvement enhanced the video sessions, which informed the survey item about the perceived benefits of caregiver participation. Second, we conducted a scoping review concerning caregivers’ support role in OT video telehealth [27]. The scoping review results that informed survey items related to caregiver roles and the types of tasks caregivers assist with during video telehealth visits.

Survey items were then evaluated for clarity and content using cognitive interviewing, an evidence-based qualitative method used to examine whether survey questions serve their intended purpose [30]. Interviews were conducted by the first author with 4 OT SMEs, in addition to the SMEs previously described, in which the first author presented the survey draft to the SMEs and asked predetermined verbal probes that focused on the clarity of items, the overall survey purpose, and whether additional items should be added. The survey was revised based on our analysis of cognitive interview data, in which the first author collated interview notes about survey items to identify those that were unclear or required further explanation. The resulting survey items were then pretested with 6 VHA OT practitioners (5 of whom were different from those who participated in cognitive interviews) to gain insights into survey functionality and time to administer, using a web-based survey link. The average survey completion time was 11 (SD 2.82) minutes.

### Survey Items

The final survey included 36 items (Multimedia Appendix 1). A total of 4 items addressed the inclusion criteria, including consent to participate, role (eg, occupational therapist or OTA), the number of completed in-home OT video encounters within the past 24 months, and the number of video encounters that involved a caregiver within the same timeframe. One of the items addressed patient factors contributing to caregiver participation in video telehealth, with a list of 12 potential factors among which respondents chose the top 5 factors. The factors included advanced age, cognitive impairments, and risk of falls. Facilitators of caregiver participation were explored through 2 items. First, participants were asked how often they used 7 facilitators of caregiver participation in video telehealth visits (including support tools; eg, national VA handouts, videos, or guides, and contacting technical support) on a 5-point Likert scale ranging from never to always. The participants were then asked to rate the effectiveness of the selected facilitators using a 5-point Likert scale that ranged from not effective to extremely effective. Adaptive questioning ensured that perceived effectiveness was collected for used facilitators. A complete list of all survey items is provided in Multimedia Appendix 1.

Data regarding caregiver assistance during video sessions were collected through 2 items in which participants were asked to rate the frequency of caregiver assistance before, during, or after video sessions for 12 technology-related tasks (eg, helping patients create or access email) and 8 clinical or nontechnological tasks (eg, offering input on patient function or details of the home and assisting with communication during sessions) on a 5-point scale ranging from never to always. The next item gathered the frequency of 9 barriers to caregiver participation in video telehealth using a 5-point scale ranging from never to always. Barriers included caregivers’ anxiety, stress, or frustration; caregivers not wanting to participate in video telehealth; and caregivers’ lack of technical skills or technical literacy. The perceived benefits of caregiver participation (eg, increased access to video telehealth) were collected through a 9-item checklist from which respondents selected all that applied.

Caregivers’ availability to assist with video telehealth was gathered via 2 items. One item addressed the frequency of instances in which video telehealth would have benefited from caregiver involvement, but caregivers were not available, using a 5-item frequency scale ranging from never to always. This was followed by a checklist item of what tended to happen if no caregiver was available to assist (eg, appointment shifted to phone). Caregivers’ relationship to patients (eg, spouse or adult child) was gathered through 1 checklist item that asked respondents to select the 3 most common relationships of caregivers who supported patient participation in video telehealth. If the participant selected paid care staff, such as home health aides, they were then prompted to provide a short description of paid care staff. Finally, participants were provided with a free-text item for any additional comments. Respondents also completed 10 practitioner demographic questions, including those on the primary VA medical center, number of years of practice, age, and practice setting. For most questions, options to select unsure or other were provided, with corresponding optional free-text boxes.

### Ethical Considerations

In accordance with institutional procedures, this project was reviewed by VA Bedford’s Institutional Review Board, which deemed the activity to be not research but quality improvement of an existing VA clinical service. Though deemed not research, the project was conducted in adherence with VA ethical and privacy protections and in accordance with the ethical standards of the relevant institutional or national bodies and consistent with the revised Helsinki Declaration [31].

### Survey Approval

Before launch, the survey was reviewed by VHA’s Organizational Assessment Sub-Committee (OASC) and Office of Labor-Management Relations (LMR) as part of standard procedures for employee surveys.

### Survey Administration

The survey was conducted between January and February 2022. VHA OT practitioners were invited to participate through an email to the VHA OT listserv, with an initial email followed by 4 follow-up reminder emails over a period of 28 days. Participants accessed the survey through a secure, anonymous link only accessible while logged into an active VA network account. As survey links were not individualized, respondents could potentially complete the survey more than once. The invitation email and survey specified that participation was voluntary, anonymous, and confidential. Respondents were able to review their answers using the back button. Study data were collected and managed using REDCap (Research Electronic Data Capture) tools hosted at VHA [32]. REDCap is a secure, web-based application designed to support data capture for research studies, providing (1) an intuitive interface for validated data entry, (2) audit trails for tracking data manipulation and export procedures, (3) automated export procedures for seamless data downloads to common statistical packages, and (4) procedures for importing data from external sources [32].

### Data Analysis

Survey data were exported from REDCap to Excel (Microsoft Corp) and summarized using frequencies and percentages. All surveys with completed eligibility questions were included in analysis; however, as item completion was not required, response numbers varied and are reported by question. Some Likert scales were collapsed (eg, combining often with always and rarely with never) for ease of presenting results. Short free-text responses were analyzed using conventional content analysis [33]. The first author (with experience in OT, telehealth, and qualitative analysis) repeatedly read responses to determine whether free-text responses differed from predetermined survey options. Concepts identified as different from predetermined survey options were then grouped into categories, which were reviewed by DEW and EEM. Rurality geocoding developed by VHA’s Office of Rural Health was used to estimate the percentage of rurality of the catchment areas associated with respondents’ primary medical center.

## Results

### Participant Characteristics

Table 1 displays the respondents’ demographics. Most respondents were female (183/226, 81%) and occupational therapists (275/286, 96.2%). Regarding ethnicity, of the 223 respondents, 18 (8.1%) identified as Hispanic or Latino, 179 (80.3%) identified as not Hispanic or Latino, and 26 (11.7%) declined to respond. Regarding race, of the 225 respondents, 4 (1.8%) identified as American Indian, Alaska Native, Native Hawaiian, or other Pacific Islander; 15 (6.7%) identified as Asian; 14 (6.2%) identified as Black or African American; 163 (72.4%) identified as White; 6 (2.7%) identified as other; and 28 (12.4%) preferred not to answer. Participants’ age, race, and gender (the data points available for VHA clinicians) aligned with those of VHA OT practitioners, according to internal VHA data. Participant demographics also closely aligned with those of OT practitioners in the United States, according to data published by the American Occupational Therapy Association (AOTA) [34]. Of note, respondents could select >1 category for race, gender, and practice setting.

**Table 1:** Respondents’ characteristics (N=286).

Demographic variables		Responses, n (%)
**Role**		
	Occupational therapist	275 (96.2)
	OT^a^ assistant	11 (3.8)
**Age (years; n=227^b^)**		
	25-34	45 (19.8)
	35-44	64 (28.2)
	45-54	64 (28.2)
	55-64	48 (21.1)
	65-74	6 (2.6)
**Race^c^ (n=225)**		
	American Indian or Alaska Native	3 (1.3)
	Asian	15 (6.7)
	Black or African American	14 (6.2)
	Native Hawaiian or other Pacific Islander	1 (0.4)
	White	163 (72.4)
	Declined to answer	28 (12.4)
	Other	6 (2.7)
**Ethnicity (n=223)**		
	Hispanic or Latino	18 (8.1)
	Not Hispanic or Latino	179 (80.3)
	Preferred not to answer	26 (11.7)
**Gender^c^ (n=226)**		
	Female	183 (81)
	Male	28 (12.4)
	Transgender or nonbinary	2 (0.9)
	Preferred not to answer	13 (5.8)
**Years of OT practice (n=232)**		
	≤5	25 (10.8)
	6-10	45 (19.4)
	11-20	56 (24.1)
	21-30	72 (31)
	>30	34 (14.7)
**Years of OT practice at VHA^d^ (n=232)**		
	≤5	85 (36.6)
	6-10	67 (28.9)
	11-20	57 (24)
	21-30	22 (9.5)
	>30	1 (0.4)
**Number of OT in-home video encounters in the last 24 months**		
	10-24	50 (17.5)
	25-99	129 (45.1)
	100+	107 (37.4)
**Frequency of OT in-home video encounters involving a caregiver in the last 24 months**		
	Rarely	41 (14.3)
	Sometimes	83 (29)
	Often	138 (48.3)
	Always	24 (8.4)
**Proportion of patients aged >65 years treated by respondent (n=232)**		
	None	0 (0)
	1%-25%	7 (3)
	26%-50%	36 (15.5)
	51%-75%	107 (46.1)
	76%-100%	82 (35.3)
**Specialty areas^c^ (n=232)**		
	Inpatient rehabilitation	51 (22)
	Outpatient rehabilitation	132 (56.9)
	Home-based primary care	43 (18.5)
	Inpatient mental health	14 (6)
	Outpatient mental health	22 (9.5)
	Skilled nursing or CLC^e^	24 (10.3)
	Homeless or HUD-VASH^f^	10 (4.3)
	Whole Health	17 (7.3)
	TREWI^g^	8 (3.4)
	Specialty	57 (24.6)
	Other	34 (14.7)

^a^OT: occupational therapy.

^b^Not all questions were required to be answered, creating variations in the sample size for each question.

^c^The respondents could select >1 answer for the questions related to race, gender, and specialty areas; therefore, the total does not add up to 100%.

^d^VHA: Veterans Health Administration.

^e^CLC: Community Living Center.

^f^HUD-VASH: Housing and Urban Development–Veterans Affairs Supported Housing.

^g^TREWI: Physical Medicine and Rehabilitation Telerehabilitation Enterprise-Wide Initiative.

Most participants (162/232, 69.8%) had >10 years of OT experience in primarily outpatient rehabilitation (132/232, 56.9%). Free-text entries for practice setting revealed that 9.1% (21/232) of the participants worked in VA’s Caregiver Support Program, a national program offering services to caregivers of eligible veterans [35]. The respondents were from 87 different VA medical centers, the catchment areas of which served a patient population that was 34% rural, on average (ranging from 0% to 98% rural).

Most respondents (189/232, 81.5%) indicated that more than half of the patients they treated were aged ≥65 years, with only 7 (3%) respondents indicating serving 1% to 25% of patients aged >65 years. None of the respondents reported not serving patients aged ≥65 years. Most respondents (179/286, 62.6%) had completed <100 in-home video encounters in the last 24 months.

### Caregiver Characteristics and Availability

Regarding the frequency of caregiver involvement in video telehealth, 56.6% (162/286) of the respondents indicated caregivers often or always participated, whereas 29% (83/286) reported caregivers sometimes participated. Regarding how often patients would have benefited from caregiver assistance with in-home video telehealth but either no caregiver was available or caregivers were not willing or able to assist, 21.6% (50/232) of the respondents reported this often or always occurred. Just under half (49.1%, 114/232) of the respondents indicated that this sometimes occurred, and 23.7% (55/232) indicated that this rarely occurred. When caregivers were not available, most indicated that the appointment was shifted to phone (157/218, 72%) or rescheduled (106/218, 48.6%).

Regarding caregivers’ role, the respondents selected the top 3 most common relationships to the patients of caregivers who participated in telehealth. Spouse was the most frequent relationship (222/235, 94.5%), followed by adult child (204/235, 86.8%) and paid care staff (90/235, 38.3%). Free-text entries describing paid care staff indicated that they were most often home health aides, with fewer reported roles for clinical staff (eg, home health nurses or home-based primary care OT practitioners). Less frequently reported relationships of caregivers who participated in video telehealth included grandchild (62/232, 26.7%); friend (24/232, 10.3%); sibling (18/232, 7.8%); and other (7/232, 3%), which, according to free-text entries, included patients’ parent, niece, or neighbor (4/232, 1.7%).

### Patient Factors Contributing to Caregiver Participation in In-Home Video Telehealth

OT practitioners were asked to identify the top 5 patient factors contributing to caregiver participation in video telehealth (Table 2). The most reported factors were patients’ lack of technical skills or technical literacy (217/285, 76.1%); cognitive impairments (eg, memory loss, executive function; 206/285, 72.3%); advanced age (173/285, 60.7%); the lack of an email address, a device (eg, laptop or smartphone), or other technological requirements (169/285, 59.3%); and hearing impairment (107/285, 37.5%). Of 285 respondents, 17 (6%) selected other, with open text entries elaborating on the given categories (eg, suicidal ideation, which is an example of a psychological factor) or indicating caregiver reasons for participation (eg, caregiver is actively involved in patient care). The lowest reported factors (other than none of the above or other) were sensory impairments (eg, sensation loss, neuropathies), which was selected by 1.4% (4/285) of the respondents, and the risk of falls, which was reported by 13.7% (39/285) of the respondents.

**Table 2:** Patient factors that contribute to caregiver participation in in-home video telehealth (n=285). Survey items were shortened for presentation; for full details, see Multimedia Appendix 1.

Patient factors that contribute to caregiver participation in video telehealth	Respondents, n (%)
Lack of technical skills or technical literacy	217 (76.1)
Cognitive impairments	206 (72.3)
Advanced age	173 (60.7)
Lack of email, device, or other technology	169 (59.3)
Hearing impairment	107 (37.5)
Motor impairments	97 (34)
Vision impairment	79 (27.7)
Communication difficulties	69 (24.2)
Psychological factors	59 (20.7)
Risk of falls	39 (13.7)
Other	17 (6)
Sensory impairments	4 (1.4)
None of the above	2 (0.7)

### Caregiver Assistance With Technological Tasks During In-Home Video Telehealth Visits

**Figure 2:**
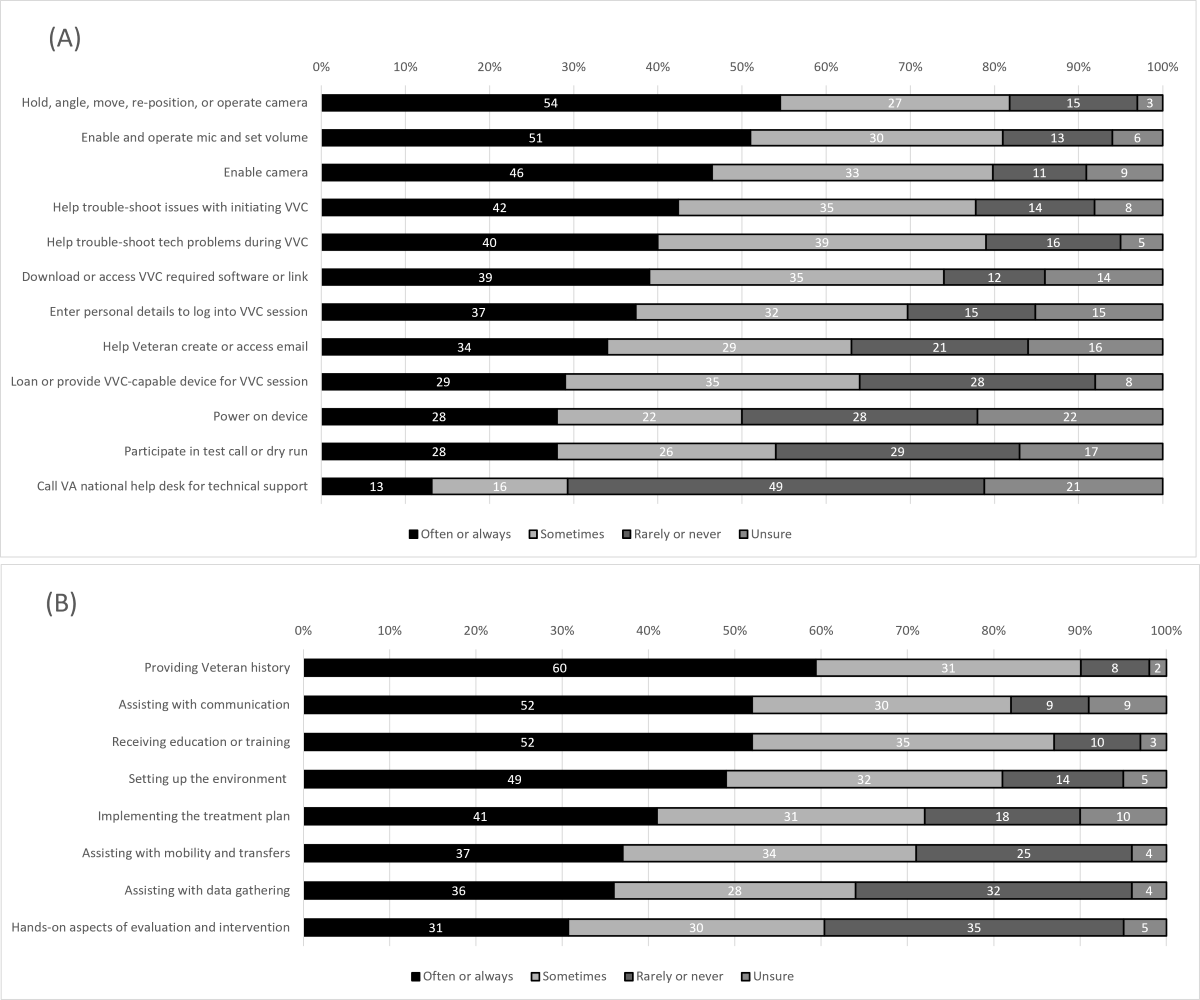
Frequency of caregivers’ assistance with technological (A) and clinical (B) tasks during video telehealth visits. In these graphs of the frequency of technological and clinical tasks with which caregivers assisted during video telehealth visits, the tasks are ordered based on the number of often or always responses. Note: Survey items were shortened for presentation; for full details, see Multimedia Appendix 1. VA: Veterans Affairs; VVC: VA Video Connect.

Respondents rated the frequency with which caregivers assisted with a list of technological tasks (Figure 2). The technological tasks with which caregivers most frequently (often or always) assisted included the following (listed in the order of frequency): holding, angling, moving, repositioning, or operating (eg, switching from front to back facing) the camera (136/250, 54.4%); enabling and operating the microphone and setting the volume (126/248, 50.8%); and enabling the camera (115/248, 46.4%). Caregivers often or always assisted with troubleshooting technology for the *initiation* of video (105/247, 42.5%) and *during* video sessions (100/248, 40.3%). Caregivers also often or always assisted with downloading or accessing the video software or link (97/249, 38.9%), entering the patient’s personal details (eg, name and home address) to log into the video session (94/250, 37.6%), helping the patient create or access email (85/250, 34%), and loaning or providing a video-capable device (72/249, 28.9%). The technological tasks with which caregivers least frequently assisted (ie, technological tasks with the highest rarely or never ratings) were participating in a test call or dry run (73/249, 29.3%) and calling the VHA’s national help desk for assistance (122/247, 49.4%).

### Caregiver Assistance With Clinical Tasks During In-Home Video Telehealth Visits

Respondents were then asked to rate the frequency of caregivers’ assistance with various clinical, nontechnological tasks (Figure 2). The tasks with the highest often or always ratings were providing history (eg, offering input on patient function or details of the patient’s home; 143/239, 59.8%), assisting with communication (eg, reminding patients of appointments or prompting, cuing, or repeating questions or instructions during sessions; 124/240, 51.7%), and receiving education and training to support patient care (124/239, 51.9%). The least frequent clinical tasks (ie, clinical tasks with the highest rarely or never ratings) were assisting with hands-on aspects of evaluation and intervention (eg, assisting with range of motion or therapeutic exercise; 83/240, 34.6%), assisting with mobility and transfers (eg, supervising or providing contact guard; 60/239, 25.1%), and data gathering (eg, taking measurements; 77/239, 32.2%).

### Facilitators of Caregiver Participation in In-Home Video Telehealth

**Figure 3:**
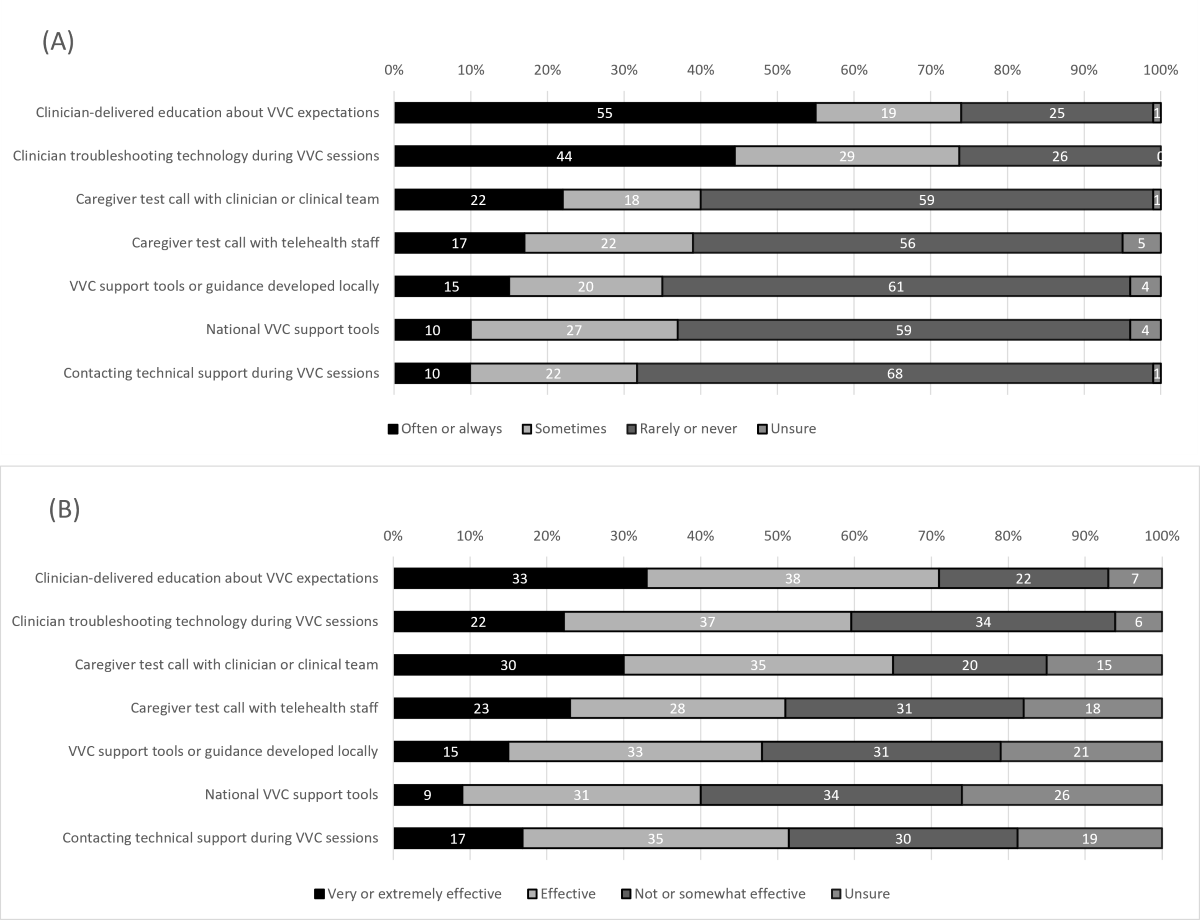
Facilitators of caregiver participation in video telehealth, including the frequency of occurrence and perceived effectiveness. Note: Survey items were shortened for presentation; for full details, see Multimedia Appendix 1. VVC: VA Video Connect.

Figure 3 displays the reported facilitators of caregiver participation in video telehealth, including the frequency of occurrence and perceived effectiveness. The facilitators with the highest often or always ratings for the frequency of occurrence were education that OT practitioners provided to caregivers about what to expect from video telehealth (152/275, 55.3%) and the OT practitioner’s own troubleshooting of technology during video telehealth visits (121/276, 43.8%). Other facilitators, such as video support tools and the use of test calls with either the OT practitioner or telehealth staff, were reported less frequently, with two-thirds (185/273, 67.8%) of respondents indicating that they rarely or never contacted technical support during video sessions. Of note, the most frequent facilitators were not always perceived as the most effective; although 43.8% (121/276) of respondents indicated often or always troubleshooting technology themselves during video telehealth visits, only 21.7% (51/235) reported their own troubleshooting as very or extremely effective. Unsure ratings for the perceived effectiveness of facilitators ranged from 6.4% to 25.6%, with the facilitators that respondents were most unsure of being video support tools (eg, national VA handouts, videos, or guides; 46/180, 25.6%) and support tools or guides that the OT practitioner or the clinical team developed locally (29/140, 20.7%). As a reminder, branching logic was such that only the respondents who used a particular facilitator (ie, selected rarely, sometimes, often, or always to the frequency item) rated its effectiveness.

### Barriers to Caregiver Participation in In-Home Video Telehealth

**Figure 4:**
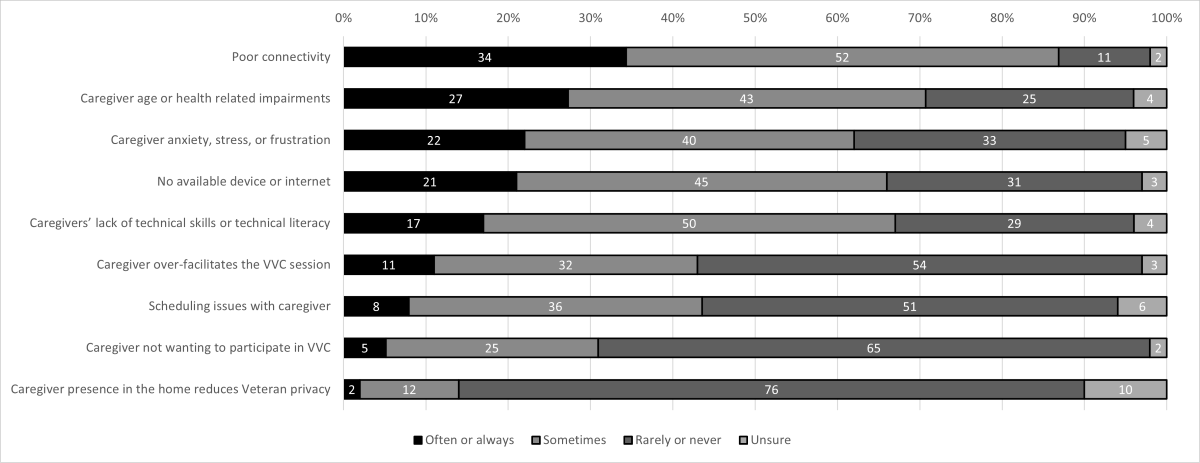
Barriers to caregiver participation in video telehealth. Note: Survey items were shortened for presentation; for full details, see Multimedia Appendix 1. VVC: VA Video Connect.

Figure 4 displays a list of reported barriers that free-text entries from the survey’s final question helped elaborate. The barriers with the highest often or always ratings were poor connectivity (80/235, 34%); caregivers’ age or health-related impairments (eg, hearing or vision loss, cognitive impairment, or mobility challenges; 64/234, 27.4%); and caregivers’ anxiety, stress, or frustration (52/235, 22.1%). Most respondents indicated that caregivers’ lack of technical skills or literacy was a barrier, with 17% (40/235) indicating that it was a barrier often or always and 50.2% (118/235) indicating that it was a barrier sometimes. Most respondents indicated rarely or never encountering barriers such as caregivers’ presence reducing patient privacy, caregivers not wanting to participate in video telehealth, or issues with scheduling.

Free-text entries underscored the impact of technological challenges for rural patients in particular, with one of the respondents noting the following: “My coverage areas are very rural. Connectivity is a problem.” Free-text entries also indicated features of the video platform as barriers, with one of the participants noting, “removing the requirement for veterans to enter their contact information into the initial screen would greatly increase veteran participation.” Free-text responses also highlighted a need for system-level supports, such as Spanish-speaking technical support, or technical support and training tailored to individual needs.

### Benefits of Caregiver Participation in In-Home Video Telehealth

Table 3 displays a list of the reported benefits of caregiver participation, with free-text entries providing further details. Benefits were gathered through a checklist item in which respondents selected all options that applied. The total number of benefits ranged from 0 to 10, with an average of 4.9 benefits per survey participant. The most frequently reported benefits were increased access to video telehealth (212/235, 90.2%) and increased collaboration with family (205/235, 87.2%). Other benefits related to the impact on care delivery, including additional information about or the verification of patient status (155/235, 66%) and increased ability to evaluate and intervene in the natural context (154/235, 65.5%). Free-text entries elaborated on the added value of caregiver involvement in video telehealth, with one of the respondents noting, “I do not think I would be able to get as much or accurate information [without caregiver assistance].”

**Table 3:** Benefits of caregiver participation in in-home video telehealth (n=235).

Benefits	Responses, n (%)
Increased access to VVC^a^ for veterans	212 (90.2)
Increased collaboration with family	205 (87.2)
Additional information about or the verification of veteran status	155 (66)
Increased ability to evaluate and intervene in the natural context	154 (65.5)
Improved engagement by veterans during visits	146 (62.1)
Decreased veteran stress	141 (60)
Improved veteran outcomes	130 (55.3)
Reduced need for formal technical support	129 (54.9)
Increased veteran compliance with the treatment plan	128 (54.5)
None of the above	1 (0.4)
Other	3 (1.3)

^a^VVC: VA Video Connect, Veteran Affairs’ videoconferencing platform.

## Discussion

### Principal Findings

Most OT practitioner respondents reported that caregivers were often or always involved in in-home OT video telehealth sessions. This, coupled with the finding that over two-thirds of the respondents served cohorts primarily aged ≥65 years, aligns with evidence endorsing caregiver assistance as facilitative to older adults’ access to video telehealth services [36,37]. Caregivers assisting with video telehealth were mostly spouses, which reflects a veteran patient population that is predominantly male and reliant on female spousal caregivers for support [38]. Adult children also frequently assisted. The finding that paid care staff (primarily home health aides) and patients’ friends also occasionally assisted patients with video telehealth underscores the need for potentially innovative solutions (eg, community-based health workers [39]) to help patients who lack familial assistance to connect with video telehealth calls.

### Patient Factors Contributing to Caregiver Participation in In-Home Video Telehealth

Regarding patient factors necessitating caregiver involvement, the primary factor was patients’ lack of technical skills or knowledge, a common barrier to older adults accessing video telehealth [39-41], followed by cognitive impairment and advanced age. The increased technical complexity of video telehealth (which exceeds plain old telephone service [POTS]) is a barrier for older adults, who lag behind younger groups in the use of the internet and videoconferencing even after the pandemic [42]. Perceived difficulties for these groups may relate to the complexity of video telehealth, which involves multiple steps such as opening a software program and enabling a camera and microphone. There are also log-in steps unique to VHA’s proprietary videoconferencing software, VVC, such as entering a phone number, address, and an emergency contact, which are meant to enhance patient safety. These additional steps may make accessing video telehealth via VVC more challenging than accessing it via commercial products such as FaceTime (Apple Inc) or Zoom (Zoom Video Communications). Evidence suggests a decreased learning curve when older adults use familiar technology [43].

Technical challenges with video telehealth may be exacerbated for those with cognitive impairment and those of a certain age. In our prior work, we interviewed patients with cognitive impairment via videoconferencing, and none of the participants were able to access videoconferencing independently [44]. Related to patient age, our finding that *advanced age* was a common factor contributing to caregiver involvement was difficult to interpret because we did not define *advanced age*. However, this finding raises concerns about the potential for ageist bias to influence clinicians’ approach to telehealth with older adults. Ageist beliefs, such as the stereotype that older adults are technophobic, can influence clinicians’ approach to telehealth, that is, to whom video telehealth is offered, and may exacerbate the digital divide [45,46]. Although age alone may be less informative than technological literacy as a contributor to the need for caregiver involvement in video telehealth, our own work and other studies suggest increased difficulty for those aged >75 years [47,48]. Age-related challenges, such as hearing and vision loss, were less frequent contributing factors, suggesting either that these challenges were less present or that they may be overcome by strategies such as increasing the volume, using headphones, or reducing visual clutter.

### Caregiver Assistance With Technological Tasks During In-Home Video Visits

Regarding technical support tasks in video telehealth, our findings reveal that caregivers assist with an array of tasks that may reflect the nature of remote delivery of OT. According to our findings, caregivers most frequently assisted with camera operation, such as holding and angling the camera. This suggests that caregivers are central to enabling clinicians to visualize the patient and the home, a key benefit of video telehealth versus other types of telehealth that lack a visual component [49]. Caregivers’ ability to assist the OT practitioner in obtaining views of the home may be particularly important for telehealth with older adults or individuals with disabilities who, because of mobility challenges or other impairments (eg, pain, fatigue, or sensory loss), may have difficulty simultaneously operating a camera and participating in clinical evaluation or intervention. Although we gathered information regarding caregiver involvement in a range of technological tasks, it should be noted that some of the lower-reported technological tasks, such as providing a device to the patient, downloading the software, and powering on the device, may have occurred before the session and therefore were not observed by the clinician. This highlights the need for a more comprehensive understanding of what caregivers do before the video session to enable patient participation. For example, clinicians could ask caregivers what steps they had to take to initiate the session and their relative ease preparing for or setting up the video session. Understanding the entire process of accessing video telehealth, including previsit steps, may help identify caregivers’ support needs.

In a related vein, the need for both clinician and caregiver technology troubleshooting during the session suggests that a test call or other preparatory sessions may go far toward reducing in-session technical challenges. However, our finding that test calls were not facilitative to caregiver-involved video sessions suggests that test calls possibly are not occurring or that they are not helpful, which warrants further study. In fact, although nearly half of the OT practitioners often or always attempted to troubleshoot technology issues during video visits, less than one-quarter felt that their attempts were very or extremely effective. This endorses the notion that solutions beyond clinician troubleshooting, such as assistance from technical support teams and caregiver training before sessions, may be required. Regarding device procurement, a key benefit of telehealth services at VHA is the provision of video-enabled tablets to patients who lack the requisite technology [50,51]. While enabling VA patients to engage, this highlights lack of telehealth technology as possibly creating disparities for patients in other health care systems [52].

### Caregiver Assistance With Clinical Tasks During In-Home Video Visits

Regarding clinical or nontechnological tasks, caregivers regularly assisted with a wide range of tasks, elaborating the potential for caregiver participation to facilitate video sessions for OT and other similarly complex clinical services. Tasks with the highest ratings related to verbal communication, such as providing patient history and reminding patients about appointments. This underscores caregivers’ frequent role as care partners, especially for older adults [53]. It also suggests the importance of communication in telehealth, particularly for older adults and others encountering communication challenges [54]. Communication challenges in video telehealth that stem from technical glitches, such as lost audio and video, can result in patients feeling less engaged. Such challenges may be reduced through a preparatory session or coaching [55]. Other barriers may relate to the nature of interpersonal communication over videoconferencing, which, although better than phone for aspects such as establishing rapport [56], may create what one team of researchers referred to as (in the context of distance learning) *transactional distance* between patients and clinicians [57], whereby patients feel less connected to care [58]. This may be exacerbated for patients whose language is different from that of the clinician [59]. Caregiver engagement by rephrasing in the patients’ language or repeating questions or information may lessen this distance.

The lowest reported clinical tasks caregivers assisted with related to hands-on aspects of evaluation and intervention, reflecting a gap in the literature about caregivers’ role in OT video sessions and in dynamic assessment more broadly. Our recent scoping review of caregivers’ support role in OT video sessions indicated that although caregivers are often mentioned as being involved in evaluation and intervention, information about the level of caregiver involvement (ie, whether they physically assisted patients or the types of assistance they provided) was generally lacking [27]. This points to a potential lost opportunity in that caregivers may be able to assist remote clinicians during video sessions by setting up the environment, operating the camera, or providing standby supervision. However, evidence for caregivers assuming such a therapist extender role during video sessions is lacking. In fact, clinical guidelines for the use of videoconferencing for performance-based assessment in general are lacking, particularly with populations contending with chronic conditions or disabilities [60,61]. A systematic review of video-delivered exercise interventions for older adults noted that although many studies cited caregiver involvement, studies did not describe what caregivers did during the video sessions [62].

More research is needed to explicate how caregivers might assist during video telehealth without increasing caregiver burden. For example, in our prior work delivering an in-home video telehealth home safety assessment to patients with dementia, which required caregivers to ambulate throughout the home while holding a portable computing device, the operation of the technology was fatiguing for some caregivers [63]. This highlights the potential negative impact of assisting during video telehealth on caregivers. Our finding that caregivers’ own health conditions or anxiety are potential barriers to their assistance during video telehealth suggests the need for guidelines regarding how to effectively partner with caregivers, particularly for tasks that might be more demanding or complex, such as assisting with mobility assessments. Caregivers’ psychosocial factors should be factored in when determining the level of assistance asked of caregivers during video telehealth, especially as some caregivers experience anxiety and social loneliness [64] or have high rates of burden [65]. This, coupled with the finding that most respondents indicated that caregivers’ lack of technical skill sometimes affected video sessions, highlights the need for caregiver-facing technical support or coaching and for an improved understanding of caregiver barriers and perspectives in general.

### Benefits of and Barriers to Caregiver Involvement

In addition to enhancing clinical care delivery, findings revealed that caregiver involvement in in-home video visits increased access to care for patients and allowed for increased collaboration with family members, especially for older patients. This aligns with evidence in which caregivers report that being involved in patients’ video visits helps them get their own questions answered [36]. It also underscores the potential for caregiver contribution in video telehealth to enhance decision-making around care transitions, an important facet of older adult care [66,67]. Findings also reveal potential challenges to caregiver involvement in video sessions, particularly among rural populations. The most frequent barrier was poor connectivity, which aligns with evidence of difficulty with Wi-Fi and internet access in rural areas [68,69]. In addition, it is important to note that challenges integrating caregivers into patient care present in brick-and-mortar settings, such as caregivers’ difficulty assisting patients with implementing care plans [70,71] or lack of knowledge about patient health conditions [72], may also be present in video visits.

Regarding the availability of assistance with video sessions, this work suggests that lack of caregiver assistance may further widen the digital divide for certain patients. The finding that it was relatively common for caregivers to not be available to assist aligns with evidence that the absence of a caregiver is a barrier to older adults’ access of video telehealth [73]. Furthermore, our finding that when caregivers were unavailable, the appointment shifted to the phone underscores the potential for patients to not receive the same quality of care if a caregiver is not available to assist. The limitations of phone to ascertaining visual information will inhibit evaluation by clinicians, such as OT practitioners, who rely on visual observation of the patient and home environment. The fact that video appointments with older patients and those from lower socioeconomic backgrounds or racial and ethnic minority groups are more likely to convert to phone [74] indicates that an unequal distribution of video telehealth may exacerbate existing health care access challenges for patients from historically marginalized populations [75].

### Limitations

This study has several limitations. VHA’s fully developed telehealth infrastructure and resources (eg, proprietary video telehealth software, national technical support hotline, dedicated technical support staff, and a tablet loaner program) may limit generalizability to health care settings that lack such resources. Nonrespondent bias may also constrain generalizability, as practitioners may have felt pressured to participate, or those with a strong interest may have been more likely to participate in the survey. Furthermore, we did not gather patient demographics or caregivers’ perspectives of video visits, knowledge that is necessary to gain a complete understanding of disparities operating within video appointments and the full extent of caregiver involvement. A more comprehensive understanding of the myriad factors involved in the video delivery of more complex services, such as OT, would enhance our ability to address digital divide issues.

### Conclusions

Although the use of video telehealth has rapidly expanded since the pandemic, digital divide issues highlight that not all individuals have equal access to the service. Patients of VHA frequently rely on caregivers to engage in video visits, particularly those who are older; who are from a rural area; or who have complex medical needs, such as dementia. Caregiver participation can enable patients to access video telehealth by providing both technical and clinical support. Such assistance is invaluable to clinical services like OT, which relies on the visualization of the home and of the patient. However, caregivers themselves may face challenges or need support in facilitating video telehealth. Furthermore, suitable assistance may need to be provided to patients who lack caregivers. By elucidating the role of caregiver support in video telehealth, including the types of tasks caregivers assist with and the benefits of caregiver participation, this study provides clinicians with considerations for how to effectively partner with caregivers to enhance older patients’ access to video telehealth.

## Appendix

Multimedia Appendix 1 Survey items.

## Abbreviations

AOTA: American Occupational Therapy Association
LMR: Office of Labor-Management Relations
OASC: Organizational Assessment Sub-Committee
OT: occupational therapy
OTA: occupational therapy assistant
POTS: plain old telephone service
REDCap: Research Electronic Data Capture
SME: subject matter expert
VA: Veterans Affairs
VHA: Veterans Health Administration
VVC: VA Video Connect
