# Supplementary material for: Caregivers’ Role in In-Home Video Telehealth: National Survey of Occupational Therapy Practitioners
Source: JMIR Rehabil Assist Technol. 2024 Mar 14;11:e52049. doi: 10.2196/52049 (PMC10979337; doi:10.2196/52049)
Supplement: Multimedia Appendix 1 [file rehab_v11i1e52049_app1.docx]

**Appendix 1.** Survey Items

*Required

Inclusion Criteria

1. *Do you agree to participate in this survey? (Yes/No)
2. *What is your role? (Occupational Therapist; Occupational Therapy Assistant; Neither)
3. *How many VVC encounters have you completed in the last 24 months? (Less than 10; 10-24; 25-99; 100 or more)
4. *In the past 24 months about how often have your VVC encounters involved a caregiver, either in set-up or completion? (Never; Rarely; Sometimes; Often; Always)

Patient Factors Contributing to Caregiver Participation

1. Which of the following Veteran factors (if any) contribute to caregiver participation in VVC? Please select the five most common factors.
   1. Advanced age
   2. Vision impairment
   3. Hearing impairment
   4. Cognitive impairments (e.g., memory loss, executive function)
   5. Motor impairments (e.g., strength, ROM, coordination, tremor)
   6. Sensory impairments (e.g., sensation loss, neuropathies)
   7. Psychological factors (e.g., stress, frustration)
   8. Communication difficulties
   9. Risk of falls
   10. Veteran lack of technical skills or technical literacy
   11. Veteran lack of email, device (e.g., laptop or smart phone), or other technological requirements for VVC
   12. Other
   13. None of the above
2. You indicated there were Veteran factors that contribute to caregiver participation in VVC not listed above. Please list the additional characteristics. (open text)

Facilitators of Caregiver Participation

1. How OFTEN have you employed the following to facilitate caregiver participation in VVC? (Never; Rarely; Sometimes; Often; Always)
   1. Caregiver test call with Telehealth (local or national help desk) prior to VVC
   2. Caregiver test call with me or someone on my clinical team prior to VVC
   3. VVC support tools, e.g., national VA hand-outs, videos or guides
   4. Support tools or guides that I or my clinical team developed locally
   5. Education that I provide to caregivers about what to expect with VVC
   6. Contacting Technical Support during VVC
   7. My trouble-shooting technology during VVC
   8. Other
2. You indicated there were facilitators to caregiver assistance that were not listed above. Please list the additional facilitators. (open text)
3. How EFFECTIVE are the following in facilitating caregiver participation in VVC? (Not effective; Somewhat effective; Effective; Very effective; Extremely effective; Unsure)
   1. Caregiver test call with Telehealth (local or national help desk) prior to VVC
   2. Caregiver test call with me or someone on my clinical team prior to VVC
   3. VVC support tools, e.g., national VA hand-outs, videos or guides
   4. Support tools or guides that I or my clinical team developed locally
   5. Education that I provide to caregivers about what to expect with VVC
   6. Contacting Technical Support during VVC
   7. My trouble-shooting technology during VVC
   8. Other
4. You noted that you or your local clinical team created tools to facilitate caregiver participation in VVC. If you are willing to share these tools, please enter your email address to be contacted. (open text)

Caregiver Assistance

1. About how often do caregivers assist in the following TECHNOLOGY-related tasks, completed before, during or after VVC? (Never; Rarely; Sometimes; Often; Always)
   1. Help Veteran create or access email
   2. Loan or provide VVC-capable device for VVC session
   3. Power on device
   4. Download or access VVC required software or link
   5. Enter personal details (e.g., name, home address) to log into VVC session
   6. Enable camera
   7. Hold, angle, move, re-position or operate (e.g., switch from front to back facing) camera during VVC
   8. Enable and operate mic and set volume
   9. Participate in test call or dry run
   10. Help trouble-shoot issues with initiating VVC
   11. Help trouble-shoot tech problems during VVC
   12. Call VA national help desk for technical support
   13. Other
2. You indicated there were technology-related tasks that would have benefited from caregiver assistance in VVC that were not listed above. Please list the additional technology-related tasks. (open text)
3. About how often do caregivers assist with the following CLINICAL (or non-technological) tasks before, during, or after VVC)? (Never; Rarely; Sometimes; Often; Always)
   1. Communication (e.g., reminding Veteran of appointment, prompting/cuing/repeating questions or instructions during session)
   2. History (e.g., offering input on Veteran function or details of home)
   3. Environmental set-up (gathering necessary objects, e.g., Theraband, measuring tape)
   4. Hands-on aspects of evaluation and intervention (e.g., assist with ROM, therapeutic exercise)
   5. Data gathering (e.g., taking measurements)
   6. Mobility and transfers (e.g., supervising, providing contact guard)
   7. Education (e.g., receiving education and training to support Veteran care)
   8. Implementing treatment plan
   9. Other
4. You indicated there were clinical (or non-technological) related tasks that would have benefited from caregiver assistance in VVC that were not listed above. Please list the additional clinical tasks. (open text)

Barriers to Caregiver Participation

1. How often have you encountered the following BARRIERS to caregiver-involved VVC? (Never; Rarely; Sometimes; Often; Always)
   1. Caregiver age or health related impairments (e.g., hearing or vision loss, cognitive impairment, mobility challenges)
   2. Caregiver anxiety, stress, or frustration
   3. Caregiver not wanting to participate in VVC
   4. Caregiver over-facilitates the VVC session
   5. Caregivers’ lack of technical skills or technical literacy
   6. No available device or internet
   7. Poor connectivity
   8. Scheduling issues with caregiver
   9. Caregiver presence in the home reduces Veteran privacy (e.g., in group treatment)
   10. Other
2. You indicated there were barriers to caregiver-involved VVC that were not listed above. Please list the additional barriers. (open text)

Benefits of Caregiver Participation

1. What are the BENEFITS of caregiver participation in VVC? Select all that apply
   1. Increased access to VVC for Veteran
   2. Decreased Veteran stress
   3. Increased ability to evaluate and intervene in natural context
   4. Increased collaboration with family
   5. Increased Veteran compliance with treatment plan
   6. Improved Veteran outcomes
   7. Additional information about or verification of Veteran status
   8. Reduced need for formal technical support
   9. Improved engagement by Veteran during visit
   10. None of the above
   11. Other
2. You indicated there were benefits of caregiver participation in VVC not listed above. Please list the additional benefits. (open text)
3. How often would the Veteran have benefited from caregiver assistance with VVC but either no caregiver was available or caregivers were not willing/able to assist? (Never; Rarely; Sometimes; Often; Always)
4. If no caregiver is available or able to assist with VVC, what tends to happen? Please select all that apply.
   1. Appointment moves to telephone
   2. Appointment moves to in-person
   3. Appointment canceled
   4. Appointment rescheduled to later date
   5. Appointment switches to FaceTime, Google Duo, or other platform
   6. Appointment goes forward (either via phone or VVC) without caregiver involvement, with NO success
   7. Appointment goes forward (either via phone or VVC) without caregiver involvement, with LIMITED success
   8. Appointment goes forward (either via phone or VVC) without caregiver involvement, with MODERATE success
   9. Appointment goes forward (either via phone or VVC) without caregiver involvement, with GREAT success
   10. Other
5. You selected ‘Other’ as an option for what happens when caregivers are not available to assist. Please describe in more detail here. (open text)

Caregiver Relationships

1. This is a list of common caregiver relationships to Veterans. For caregivers who support Veteran participation in VVC, what are caregivers’ 3 most common relationships to Veterans?
   1. Spouse
   2. Adult child
   3. Sibling
   4. Grandchild
   5. Friend
   6. Neighbor
   7. Paid care staff, such as home health aide
   8. Other
2. You indicated “paid care staff” as 1 of 3 most frequency relationship of caregivers supporting Veteran participation in VVC. Please provide a short description of paid care staff (e.g., home health aide). (open text)
3. You indicated “other” as 1 of 3 most frequency relationship of caregivers supporting Veteran participation in VVC. Please provide a short description of this relationship (open text)

OT Demographics

1. How many years you been a practicing OT? (5 years or less; 6-10; 11-20; 21-30; More than 30)
2. How many of those years you been a VA OT? (5 years or less; 6-10; 11-20; 21-30; More than 30)
3. Primary VAMC with which you are affiliated (List of VA Medical Centers
4. What percentage of the Veterans you treat are over 65 years of age? (None; 1-25%; 26-50%; 51-75%; 76-100%)
5. What are your practice settings/specialty areas? Select all that apply
   1. Inpatient Rehabilitation
   2. Outpatient Rehabilitation
   3. Home Based Primary Care
   4. Inpatient Mental Health
   5. Outpatient Mental Health
   6. Skilled Nursing/CLC
   7. Homeless/HUD-VASH
   8. Whole Health
   9. Telerehab Enterprise Wide Initiative (TREWI)
   10. Specialty area, e.g., TBI, spinal cord injury, driver rehabilitation
   11. Other
6. Please describe your specialty area (open text)
7. You described your practice setting as “other.” Please describe your practice setting (open text)
8. What is your age? (18-24 years old; 25-34 years old; 35-44 years old; 45-54 years old; 55-64 years old; 65-74 years old; 75-84 years old; Older than 85)
9. What is your race? Select all that apply. (American Indian or Alaska Native; Asian; Black or African American; Native Hawaiian or Other Pacific Islander; White; Declined to answer; Other)
10. What is your ethnicity? (Hispanic or Latino; Not Hispanic or Latino; Prefer not to answer)
11. What is your gender? Select all that apply. (Male; Female; Transgender or Non-binary; Prefer not to answer)
12. Is there anything else you would like to tell us about caregivers and VVC?
